# Study on the relationship between medical staff’s knowledge, attitudes, and practices regarding medical waste classification and personality traits

**DOI:** 10.3389/frhs.2025.1609584

**Published:** 2025-09-16

**Authors:** Jinyan Wang, Meifeng Liu, Deyu Wang

**Affiliations:** ^1^ Shandong Urban Construction Vocational College, Jinan, China; ^2^ Division of Gastrointestinal Surgery, Ward 2, Olympic Branch, Shandong Provincial Hospital Affiliated to Shandong First Medical University, Jinan, China

**Keywords:** medical waste, health, knowledge, attitude, practice, personality (with five-factor model assessment), health personnel

## Abstract

**Aim and objectives:**

This study aims to assess healthcare workers’ medical waste management knowledge, attitudes, and practices (KAP) and the influencing Factors; The study objectives are to explore the association between medical staff's personality traits and their KAP toward medical waste classification.

**Methods:**

A self-designed questionnaire assessing medical staff's knowledge, attitudes, and Practices toward medical waste classification, along with the Ten-Item Personality Inventory - Chinese version (TIPI-C), was administered to 420 nurses and doctors at a hospital in China. Group comparisons were performed using t-tests and ANOVA. Correlations between medical staff's knowledge and attitudes toward medical waste classification and TIPI-C were analyzed using Spearman's correlation. Influencing factors were examined through multiple stepwise regression analysis.

**Results:**

This study assessed the knowledge, attitudes, and practices (KAP) of medical waste classification among 420 healthcare professionals (214 nurses, 206 doctors) in a large Chinese hospital. The overall scores were 8.70 ± 1.63 for knowledge, 18.54 ± 3.11 for attitudes, and 24.20 ± 4.94 for practices. Nurses demonstrated significantly higher KAP levels than doctors across all domains (*P* < 0.05). Female staff outperformed males in knowledge (*β* = −0.162, *p* = 0.002), attitudes (*β* = −0.266, *P* < 0.001), and practices (*β* = −0.212, *P* = 0.002). Longer working experience was positively associated with knowledge (*β* = 0.113, *P* = 0.019). Higher education was also a positive predictor of knowledge (*β* = 0.132, *P* = 0.007). Among personality traits, openness showed a significant positive correlation with attitudes (*r* = 0.187, *P* < 0.01) and was a predictor of both attitudes (*β* = 0.160, *P* = 0.017) and practices (*β* = 0.154, *P* = 0.025) in regression analysis.

**Conclusions:**

This study revealed moderate to high levels of knowledge, attitudes, and practices (KAP) regarding medical waste classification among healthcare professionals, with nurses and female staff demonstrating significantly higher KAP scores. Key influencing factors identified include occupation, gender, years of experience, education level, and the personality trait of openness. These findings highlight the need for targeted, role-specific training programs to enhance compliance and safety in medical waste management. In addition to training, policy implications should include the integration of medical waste management into regular performance assessments and accountability mechanisms. Furthermore, fostering a culture of openness and continuous improvement through institutional support and feedback systems is recommended to sustain positive behavioral change.

## 1 Backgrounds

The environmental impact of medical waste has emerged as a critical global challenge, given its highly polluting nature and associated public health risks (1). Globally, contemporary medical waste classification systems demonstrate significant shortcomings, particularly in sorting accuracy (58% in low- and middle-income countries (2, 3), which constitutes a major barrier to effective management. These findings not only highlight the urgent need for standardised categorisation protocols but also drive international initiatives to optimise waste disposal infrastructures. This has led to increased international focus on improving waste management and disposal systems. In 2003, the State Council of China introduced the Regulations on the Management of Medical Waste, emphasizing that the proper handling of medical waste is crucial for the safety and health of people both in China and worldwide (4). With the growing public awareness of health issues, medical waste disposal has become a part of the research and management agenda at various levels of government. Many countries and regions around the world have implemented a series of measures that provide clear regulations on the classification, disinfection, storage, and disposal of medical waste. By contrast, research and management in China remain in the early stages, with widespread problems of improper medical waste segregation in hospitals (5). The study by Liu and Yao revealed that the rate of incorrect medical waste segregation in some hospitals reached 30%–35% (6). This not only increases the cost of medical waste disposal but also causes significant harm to both the environment and human health. Improving the ability of hospital staff to correctly and effectively classify medical waste can significantly reduce environmental pollution and mitigate the direct and indirect harm to human health (7, 8). Personality traits, which are psychological tendencies that lead to consistent patterns in an individual's practice, play a key role in influencing actions, work, lifestyle, and health (9–11). Several studies have confirmed the association between the Knowledge-Attitude-Practice (KAP) model and personality traits (12). Partial academic evidence also indicates that the Five-Factor Model (FFM) demonstrates significant predictive validity for environmental improvement behaviors, with observed correlations between conscientiousness/openness traits and accuracy in waste segregation practices (13). However, the relationship between knowledge, attitudes, and Practices (KAP) regarding medical waste sorting and personality traits warrants further exploration. This study aims to assess the association between medical waste classification knowledge, attitudes, and practices and personality traits, providing valuable theoretical insights for policy development and management strategies. The results are presented below.

## 2 Participants and methods

### 2.1 Study participants

From January to October 2024, a convenience sampling method was employed to select 420 nurses and doctors from a large general hospital in Shandong Province, China, with over 7,000 staff members. The inclusion criteria were: 1) No cognitive impairment; 2) Informed consent and voluntary participation in the survey. The exclusion criterion was non-cooperation with the survey. The sample size was calculated using the formula *n* = *Z*^2^_1−*a*/2_*p*(1−*p*)/*d*^2^ (14). Where *n* is the sample size, *Z*_1−*a*/2_ is the standard normal variate (1.96 at a 5% level of significance); *P* = 0.58 (15) represents the awareness rate of medical waste classification knowledge, the qualification rate of attitudes, or the implementation rate of practices; and *d* *=* 0.05 (14), refers to precision or absolute error. The maximum sample size will be calculated by substituting the highest value among the knowledge awareness rate, attitude qualification rate, or practice implementation rate of medical waste classification found in the literature into the aforementioned formula. This yielded a calculated sample size of *n* = 374. Furthermore, since this study requires multiple linear regression analysis, a common rule of thumb for sample size determination applies. Rule of thumb: A minimum of 10–20 observations per independent variable is required. This study intends to include 10 independent variables (e.g., gender, age, work experience, education level, and five personality trait dimensions). Thus, the required sample size would range from at least 10 × 10 = 100 to 10 × 20 = 200 observations. Accounting for a 10% anticipated rate of invalid responses, the minimum required sample size was calculated as 411 participants to ensure adequate statistical power. The final sample size for the study was 420.

### 2.2 Materials and methods

#### 2.2.1 Research tools

##### 2.2.1.1 General information questionnaire for medical staff

The questionnaire was a self-designed questionnaire that includes gender, age, years of experience, and education.

##### 2.2.1.2 Medical staff's knowledge, attitudes, and practices regarding medical waste classification

A survey on the knowledge, attitudes, and behaviors of medical staff regarding medical waste classification was designed based on a review of relevant literature (16, 17). The expert panel, which consisted of two nursing department heads, four hospital infection management experts, and two university professors in public health, collaboratively designed the questionnaire. The questionnaire covers three key areas: knowledge, attitudes, and Practices, with a total of 23 items. The knowledge dimension includes 12 items, such as the hazards of medical waste, types of medical waste, and the specific classification of different types of medical waste. The questions include both multiple-choice and single-choice formats to assess the accuracy of responses. The attitude dimension includes five items, such as “I believe correct medical waste classification is very important,” “I believe it is an inevitable trend in medical development,” “I believe correct classification helps improve patient satisfaction,” “I find proper classification difficult,” and “I believe proper classification is not relevant to me.” Items were measured using a 5-point Likert scale ranging from 1 (“Strongly Disagree”) to 5 (“Strongly Agree”), yielding a potential total score range of 5–25 points per instrument, where higher scores indicate stronger agreement. Scores between 5 and 15 indicate a low level, 16–20 indicate a medium level, and 21–25 indicate a good level. The practice dimension includes six items related to the classification of common medical waste types, such as chemical, infectious, pathological, pharmaceutical, and injury-related waste. This section also uses a 5-point Likert scale: “More than 10 times/week” = 5 points, “6–9 times/week” = 4 points, “3–5 times/week” = 3 points, “1–2 times/week” = 2 points, and “Occasionally” = 1 point, with a total score range of 6–30 points. Scores between 5 and 15 indicate a low level, 16–20 indicate a medium level, and 21–30 indicate a good level. A pre-survey was conducted with 30 medical staff members. The Cronbach's *α* coefficients for the total questionnaire, as well as for the knowledge, attitude, and practice sections, were 0.814, 0.824, 0.828, and 0.847, respectively. The test-retest reliability was 0.854, 0.849, 0.837, and 0.843. Validity was assessed using the Kaiser-Meyer-Olkin (KMO) method, and the KMO value for this study was 0.836, with a *p*-value of less than 0.05, indicating good validity.

##### 2.2.1.3 Ten-Item personality inventory-Chinese version, TIPI-C) (18)

The instrument comprises five distinct subscales measuring the following core personality dimensions: Extraversion, Agreeableness, Conscientiousness, Neuroticism, and Openness to Experience, with a maximum possible total score of 70. Extraversion: Characterized by sociability, talkativeness, assertiveness, and high levels of emotional expressiveness. Individuals high in extraversion tend to seek stimulation in the company of others. Agreeableness: Reflects individual differences in concern for social harmony. It includes traits like trust, altruism, kindness, affection, and other prosocial behaviors. Conscientiousness: Refers to tendencies toward self-discipline, dutifulness, competence, thoughtfulness, and achievement-striving (against measures or outside expectations). Higher scores indicate a stronger presence of the trait within the subscale. Neuroticism: This dimension refers to the tendency to experience negative emotions such as anger, anxiety, or depression. It also relates to emotional instability and susceptibility to stress. Openness to Experience: This trait is characterized by appreciation for art, emotion, adventure, unusual ideas, imagination, curiosity, and variety of experience. It reflects cognitive flexibility and creativity. The internal consistency reliability coefficients (Cronbach's *α*) for the respective scales in this study were computed as 0.782, 0.803, 0.812, 0.794, and 0.843, demonstrating acceptable to good reliability across all measures.

#### 1.2.2 Survey method

The researchers recruited 425 doctors and nurses from 10 departments across the entire hospital district (e.g., Gastrointestinal Surgery, Gastroenterology, Cardiology, etc.) to participate in the survey via Wenjuanxing. After excluding questionnaires with errors and incomplete responses, 420 valid questionnaires were retained.

#### 1.2.3 Statistical methods

All statistical computations were executed in SPSS 25.0. Descriptive statistics comprised frequency distributions (*n*, %) for categorical variables and mean ± standard deviation for continuous variables. Intergroup comparisons were performed using independent samples t-tests for pairwise comparisons and one-way analysis of variance (ANOVA) for multi-group comparisons. Bivariate associations between personality traits and medical waste-related knowledge, attitudes, and practices were examined using Spearman's rank-order correlation. Subsequently, multiple stepwise linear regression analysis was employed to identify significant predictive factors. The stepwise regression analysis employs three key screening criteria: (1) Statistical significance based on *p*-values, with an entry criterion of *P* < 0.05 for including variables and a removal criterion of *P* > 0.10 for excluding them; (2) Information criteria prioritizing higher adjusted *R*^2^ values to prevent artificial inflation from additional variables while selecting the optimal model; (3) Auxiliary diagnostics including Variance Inflation Factor (VIF) to detect multicollinearity, where VIF values exceeding 5 or 10 indicate severe multicollinearity issues. The statistical significance threshold was set at *P* < 0.05. The total scores of knowledge, attitudes, and practices regarding medical waste classification were taken as the dependent variables. Demographic data and TIPI-C were used as independent variables. These independent variables were assigned as dummy variables and all were incorporated into a comprehensive multiple regression model. The assignment method for the independent variables is shown in Table 1.

**Table 1:** Assignment of independent variables to the factors influencing medical staff's knowledge, attitudes and practices on medical waste classification.

Items	Assignment method
Gender	Female = 1; Male = 2
Years of working experience (years)	<2 = 1; 2∼5 = 2; 6∼10 = 3; >10 = 4
Occupation	Nurse = 1;Docto*r* = 2
Age (years)	<30 = 1; 30∼50 = 2; >50 = 3
Education	Associate/Undergraduate = 1; Master/Doctor and above = 2
Knowledge, attitudes, and practices regarding medical waste classification	Original value
Ten-item personality inventory-Chinese version (extraversion, agreeableness, neuroticism, conscientiousness, openness)	0∼7 = 0;8∼14 = 1

Dummy variables were used in the multiple linear regression analysis for TIPI-C. Specifically a score of 0–7 on each dimension was assigned a value of 0, and a score of 8–14 on each dimension was assigned a value of 1.

## 3 Results

### 3.1 Medical staff's knowledges, attitudes, and practices regarding medical waste classification

**Figure 1:**
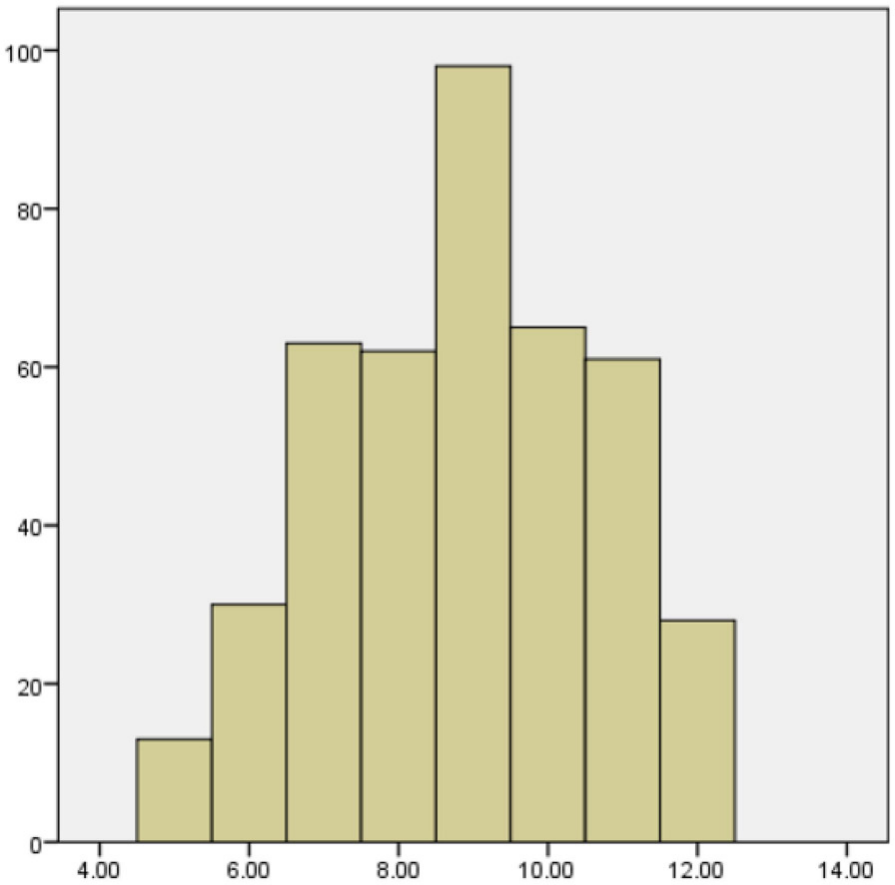
Histogram of knowledge scores on medical waste segregation.

**Figure 2:**
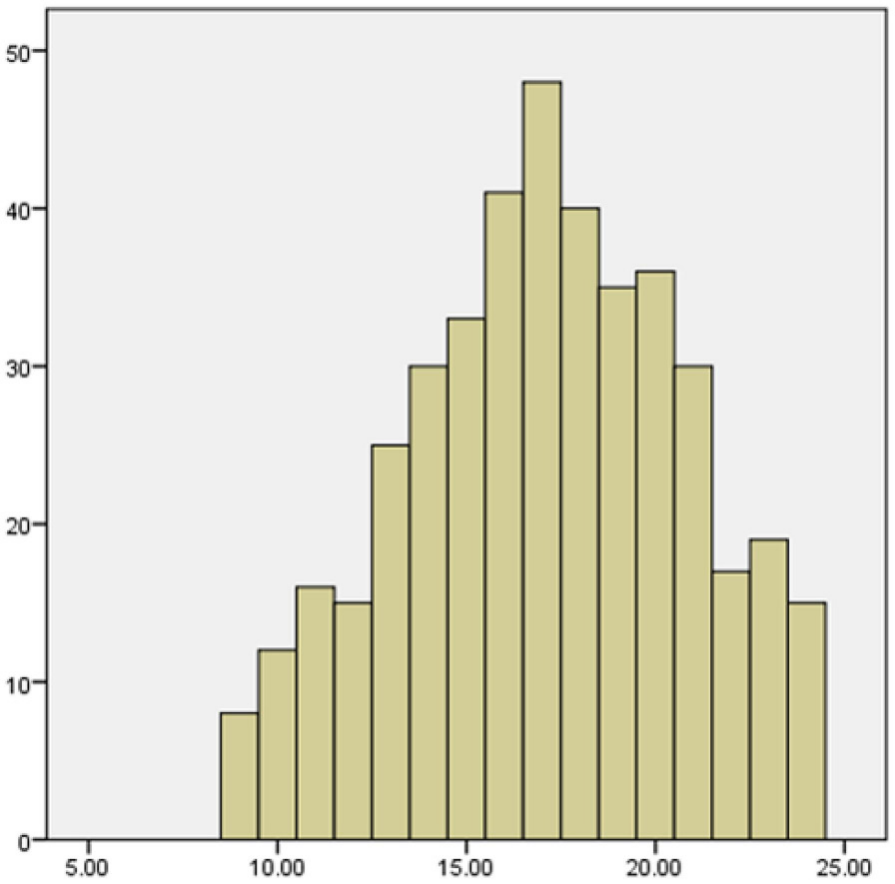
Histogram of attitude scores on medical waste segregation.

**Figure 3:**
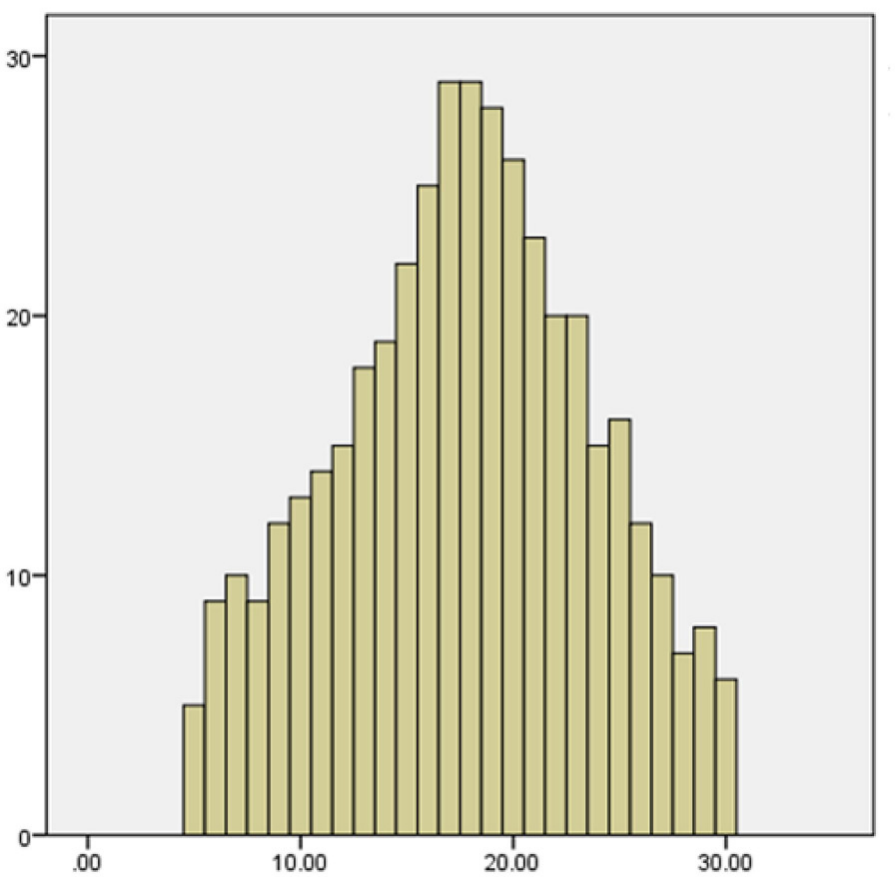
Histogram of practice scores on medical waste segregation.

Based on a study of 420 medical staff (214 nurses, 206 doctors), the overall scores for medical waste classification were 8.70 ± 1.63 (Knowledge), 18.54 ± 3.11 (Attitude), and 24.20 ± 4.94 (Practice). Nurses consistently outperformed doctors across all domains, and female staff scored significantly higher than males (*P* < 0.05). Staff with 6–10 years of experience showed the highest knowledge and attitude scores. Age and education level did not significantly influence the results(see Tables 2–4). The histograms show that the distributions of medical staff's knowledge, attitudes, and practices regarding medical waste classification are approximately normal, as shown in Figures 1–3.

**Table 2:** Score of medical staff's knowledge on classification of medical waste (*N* = 420) (score, $\bar{\chi }\pm s$).

Items	Nurse (*n* = 214)	Doctor (*n* = 206)	Total (*N* = 420)
Gender			
Female	9.25 ± 1.38	8.54 ± 1.65	9.03 ± 1.51
Male	8.59 ± 1.85	8.03 ± 1.64	8.15 ± 1.70
*t*	2.349	2.184	5.525
*P*	0.020	0.030	0.00
Age (years)			
<30	9.09 ± 1.48	8.42 ± 1.78	8.76 ± 1.66
30∼50	9.22 ± 1.49	8.28 ± 1.54	8.71 ± 1.59
>50	9.13 ± 1.48	7.82 ± 1.71	8.62 ± 1.69
*F*	0.169	1.651	0.204
*P*	0.844	0.194	0.816
Years of working experience (years)			
<2	8.83 ± 1.54	7.70 ± 1.37	8.39 ± 1.57
2∼5	9.22 ± 1.59	8.54 ± 1.63	8.77 ± 1.65
6∼10	9.59 ± 1.17	8.05 ± 1.64	8.90 ± 1.59
>10	9.09 ± 1.49	8.45 ± 2.14	8.89 ± 1.73
*F*	2.724	3.066	2.270
*P*	0.045	0.029	0.080
Education			
Associate/Undergraduate	9.03 ± 1.50	8.00 ± 1.57	8.66 ± 1.60
Master/Doctor and above	9.44 ± 1.39	8.40 ± 1.70	8.77 ± 1.67
*t*	−1.895	−1.717	−0.691
*P*	0.059	0.088	0.490

The overall knowledge score of the entire sample was 8.70 ± 1.63.

**Table 3:** Score of medical staff's attitudes on classification of medical waste (*N* = 420) (score, $\bar{\chi }\pm s$).

Items	Nurse (*n* = 214)	Doctor (*n* = 206)	Total (*N* = 420)
Gender			
Female	19.46 ± 2.23	18.60 ± 3.02	19.19 ± 2.53
Male	16.69 ± 4.60	17.63 ± 3.38	17.43 ± 3.67
*t*	3.334	2.213	5.789
*P*	0.000	0.035	0.000
Age (years)			
<30	18.61 ± 3.02	18.22 ± 3.41	18.42 ± 3.21
30∼50	19.48 ± 2.99	18.10 ± 3.05	18.72 ± 3.10
>50	18.92 ± 2.49	17.47 ± 3.58	18.36 ± 3.03
*F*	1.844	0.675	0.597
*P*	0.161	0.510	0.551
Years of working experience (years)			
<2	18.93 ± 2.84	17.00 ± 3.53	18.17 ± 3.26
2∼5	19.52 ± 2.59	18.38 ± 3.12	18.76 ± 2.99
6∼10	19.61 ± 3.45	18.74 ± 3.44	19.22 ± 3.45
>10	18.02 ± 2.24	17.15 ± 2.25	17.75 ± 2.26
*F*	3.007	3.173	3.695
*P*	0.000	0.025	0.012
Education			
Associate/Undergraduate	18.97 ± 2.88	17.99 ± 3.40	18.62 ± 3.11
Master/Doctor and above	19.20 ± 2.89	18.04 ± 3.18	18.45 ± 3.13
*t*	−1.524	−0.114	0.554
*P*	0.601	0.909	0.580

The overall attitude score of the entire sample was 18.54 ± 3.11.

**Table 4:** Score of medical staff's practice on classification of medical waste (*N* = 420) (score, $\bar{\chi }\pm s$).

Items	Nurse (*n* = 214)	Doctor (*n* = 206)	Total (*N* = 420)
Gender			
Female	24.96 ± 4.65	24.75 ± 4.90	24.89 ± 4.72
Male	22.47 ± 4.04	23.16 ± 5.34	23.02 ± 5.09
*t*	2.844	2.159	3.808
*P*	0.005	0.032	0.000
Age (years)			
<30	24.07 ± 4.85	23.97 ± 3.93	24.02 ± 4.41
30∼50	23.35 ± 4.65	24.23 ± 6.11	24.74 ± 5.51
>50	24.08 ± 4.28	22.34 ± 4.26	23.41 ± 4.33
*F*	1.955	1.893	2.487
*P*	0.144	0.153	0.084
Years of working experience (years)			
<2	23.93 ± 2.84	22.92 ± 4.29	23.58 ± 4.72
2∼5	24.52 ± 2.59	24.72 ± 5.76	24.70 ± 5.19
6∼10	24.61 ± 3.45	24.24 ± 3.88	24.75 ± 4.66
>10	23.02 ± 2.24	20.50 ± 5.47	23.41 ± 5.01
*F*	3.007	4.450	2.053
*P*	0.031	0.005	0.106
Education			
Associate/Undergraduate	24.64 ± 4.68	23.50 ± 5.20	24.23 ± 4.90
Master/Doctor and above	24.45 ± 4.57	24.01 ± 5.23	24.16 ± 5.00
*t*	0.272	−0.687	0.131
*P*	0.786	0.493	0.896

The overall practice score of the entire sample was 24.20 ± 4.94.

### 3.2 Correlation between medical waste classification knowledge, attitudes, and practices and the Chinese version of the big five personality traits

Openness is significantly positively correlated with knowledge, attitudes, and practices (KAP) regarding medical waste classification, while Extraversion shows a significant positive correlation with waste classification practices (see Table 5).

**Table 5:** Correlation between medical staff's knowledge, attitudes and practices on medical waste classification and personality (*r*).

Items	Knowledge	Attitudes	Practices
Extraversion	0.022	0.082	0.140**
Agreeableness	−0.093	−0.048	0.037
Neuroticism	0.049	0.053	0.078
Conscientiousness	−0.038	−0.031	0.013
Openness	0.101*	0.187**	0.100*

Analyzed using Spearman's correlation analysis; *r* = Spearman's correlation coefficient.

∗*P* < 0.05;

∗∗*P* < 0.01.

### 3.3 Factors influencing medical staff's knowledge, attitudes, and practices regarding medical waste classification

After assigning values to the variables according to Table 1, all independent variables and the dependent variable were introduced into a multiple linear regression analysis. The results are shown in Table 6. The multiple stepwise regression analysis revealed that gender was a consistent and significant predictor across all three domains of medical waste management, with female staff demonstrating higher levels of knowledge (*β* = −0.162, *P* = 0.002), more positive attitudes (*β* = −0.266, *P* < 0.001), and better practices (*β* = −0.212, *P* = 0.002). Knowledge: Females scored 9.03 ± 1.51 on average, while males scored 8.15 ± 1.70, with a mean difference of 0.88 ± 1.61 (*t* = 5.525, *P* < 0.001). Attitudes: The mean attitude score for females was 19.19 ± 2.53, compared to 17.43 ± 3.67 for males, with a mean difference of 1.76 ± 3.10 (*t* = 5.789, *P* < 0.001). Practices: The mean practice score for females was 24.89 ± 4.72, while for males it was 23.02 ± 5.09, with a mean difference of 1.87 ± 4.91 (*t* = 3.808, *P* < 0.001). Knowledge was additionally influenced by occupation (*β* = 0.227, *P* < 0.001) [The mean knowledge score for nurses was 9.25 ± 1.38, compared to 8.54 ± 1.65 for physicians, with a mean difference of 0.71 ± 1.52 (*t* = 2.349–2.184, *P* < 0.05)], years of experience (*β* = 0.113, *P* = 0.019) (For example, those with 6–10 years of experience had a mean knowledge score of 8.90 ± 1.59, while those with <2 years scored 8.39 ± 1.57), and education level (*β* = 0.132, *P* = 0.007) [the mean knowledge score for Master/Doctor holders was 8.77 ± 1.67, while for associate/undergraduate holders it was 8.66 ± 1.60, and the group difference was not statistically significant (*t* = −0.691, *P* = 0.490)]. The personality trait openness positively predicted both attitudes (*β* = 0.160, *P* = 0.017) and practices (*β* = 0.154, *P* = 0.025). All variance inflation factor (VIF) values remained below 2.1, indicating the absence of multicollinearity among the predictors.

**Table 6:** Results of multiple stepwise regression analysis of factors influencing knowledge, attitudes and practices.

Items	SE	*β*	*t*	*P*	VIF
Knowledge					
Constant term	0.637	–	11.424	0.000	
Gender	0.177	−0.162	−3.093	0.002	1.304
Years of working experience	0.077	0.113	2.348	0.019	1.095
Occupation	0.178	0.227	4.161	0.000	1.415
Education	0.160	0.132	2.711	0.007	1.129
Attitudes					
Constant term	1.240	–	−15.503	0.000	
Gender	0.345	−0.266	−4.978	0.000	1.304
Openness	0.552	0.160	2.387	0.017	2.062
Practices					
Constant term	1.192	–	12.608	0.000	
Gender	0.554	−0.212	−3.907	0.002	1.304
Openness	0.887	0.154	2.257	0.025	1.978

## 4 Discussions

### 4.1 Status of medical staff's knowledge, attitudes, and practices in medical waste classification

Results indicated the average score o for medical staff's knowledge of medical waste classification (8.70 ± 1.63), reflecting lower levels. Further analysis revealed that individuals with lower scores were predominantly male and had fewer than two years of work experience. Based on the accumulated evidence, we conclude that the main weakness in medical staff's knowledge of medical waste classification lies in the specialised knowledge of medical waste management. This could be attributed to insufficient theoretical knowledge and limited training on waste classification. It is difficult for staff to correctly implement medical waste classification (19) without adequate theoretical support. This highlights the need for hospital administrators to focus on enhancing clinical training, particularly in the area of hospital infection management theory. A series of lectures should be organised to address related issues, with the use of multimedia, such as large screens and WeChat, to aid education and publicity (20). Studies have shown that systematic training on the classification of medical and household waste can significantly promote waste management in healthcare institutions (21, 22). Visual guidance systems can enhance medical waste segregation accuracy. This involves positioning instructional labels above collection bins, incorporating both graphical cues and concise textual reminders (23).

The statistical analyses consistently indicate that the medical staff's attitude towards medical waste classification scored an average of (18.54 ± 3.11), which is regarded as a good level. Bektaş Y (24) suggested that beliefs determine attitudes and motivation, meaning that only with a strong belief in the importance of medical waste classification can healthcare workers properly implement the task. In this study, nurses displayed a more positive attitude towards medical waste classification, with their scores typically higher than those of doctors. This could be attributed to the hospital's three-tier infection control quality management system. The Infection Control Department, Nursing Department, and Occupational Health Department are responsible for primary quality management control, with head nurses overseeing secondary control and infection nurses managing the third tier. This system has further reinforced the nurses' understanding of infection control, making them more aware of its significance. As a result, nurses tend to adopt a more meticulous and conscientious approach to medical waste classification. In contrast, doctors, particularly those undergoing training, face significant challenges in receiving training on the theory and principles of medical waste classification due to their frequent movement between departments.

The results of this study revealed that the medical staff's correct practice in classifying medical waste scored an average of (24.20 ± 4.94), which is considered a moderate level. Further analysis indicated that those with lower scores were predominantly junior nurses and doctors, which aligns with the findings of related studies (25, 26). The analysis suggests that pharmaceutical and injury-related medical waste are relatively easy to identify, whereas pathological, chemical, and infectious waste are more difficult to recognise (27, 28). This may be due to the fact that these three types of waste are more specialised, making them harder to identify for newly trained or less experienced medical staff. In comparison, pharmaceutical and injury-related medical waste are easier to distinguish, and are therefore more straightforward to classify. This highlights the need for hospital administrators to focus on training and assessment in the classification of these more challenging types of waste.

### 4.2 Factors influencing medical staff's knowledge, attitudes, and practices regarding medical waste classification

The multiple regression analysis on knowledge regarding medical waste classification revealed several significant predictors. Gender (*β* = −0.162, *P* = 0.002) was a significant factor, with female healthcare workers demonstrating higher knowledge scores. This is consistent with previous studies suggesting that gender may influence exposure to training and engagement in waste management practices (29). Years of working experience (*β* = 0.113, *P* = 0.019) also positively influenced knowledge, indicating that longer tenure is associated with greater familiarity with waste classification protocols, likely due to accumulated practical experience (30). Occupation (*β* = 0.227, *P* < 0.001) was another strong predictor, with nurses showing higher knowledge levels than doctors (29). This may be attributed to nurses' more direct and frequent involvement in daily waste handling tasks (Al-Emad, 2021). Educational level (*β* = 0.132, *P* = 0.007) was positively associated with knowledge, supporting the notion that higher education often correlates with better comprehension of guidelines and regulations (31). This study found that female healthcare workers, those with higher educational levels, nurses, and healthcare workers with more than two years of experience respectively possess a more comprehensive understanding of medical waste classification knowledge.

Multiple stepwise regression analysis identified gender and openness as statistically significant predictors of medical staff's attitudes toward medical waste classification (*P* < 0.05). This suggests that employees with open personality traits and female medical staff tend to have more confidence in their work, maintaining an organized and focused approach. Bivariate correlation analysis revealed a significant positive association between openness personality trait and positive attitudes toward medical waste classification (*P* < 0.01). Research has demonstrated that openness as a personality trait is characterised by imagination, critical thinking, and a strong set of values (32, 33).

The results from the multiple stepwise regression analysis indicated that gender and openness are the primary factors influencing medical staff's practice in medical waste classification (*P* < 0.05). Both extraversion and openness personality traits showed significant positive correlations with proper medical waste categorization behavior (all *P* < 0.01). According to the Knowledge-Attitude- Practice (KAP) theory, comprehensive theoretical knowledge and correct beliefs serve as the driving forces for practice change, with knowledge and attitudes ultimately determining practice (34). In this study, gender and openness were found to be influential factors not only for knowledge and attitudes but also for practice. This suggests that female medical staff and those with an open personality are more likely to improve their medical waste classification practice more quickly.

## 5 Conclusion

The research indicates healthcare professionals' medical waste classification competencies demonstrate intermediate proficiency across knowledge, attitudes and practices, revealing considerable scope for enhancement. While specialist knowledge constitutes the primary modifiable determinant of implementation efficacy, the incorporation of personality metrics yields intriguing insights. Although conscientiousness showed no significant correlation with segregation practices in this study, openness to experience emerged as a potentially valuable predictor of innovative classification approaches. These findings, albeit limited by the cross-sectional design and single-institution sampling, suggest personality-informed training could address both fundamental compliance and progressive improvement in waste management systems.

## 6 Recommendations

This study has several limitations: unadjusted social desirability bias (e.g., overestimation of knowledge through self-reported questionnaires) potentially underestimates correlations between neuroticism and non-compliant behaviors (e.g., improper sharps disposal), while the single-hospital setting restricts variability in organizational cultures, management protocols, and staff demographics, affecting the external validity of personality-waste practice relationships. Future research should incorporate unobtrusive behavioral observation methods (e.g., systematic waste bin audits) to enhance validity and retain conscientiousness assessment due to its predictive value for procedural adherence. Caution is required when extrapolating findings to institutions with differing infrastructure, policy standards, or regional regulations.

Hospital management should strengthen infection control training through mandatory competency evaluations integrating (a) humanistic competencies, (b) waste classification principles, and (c) operational proficiency benchmarks. A dual-phase training approach is recommended: Phase 1 delivers standardized compliance modules to build core competencies, while Phase 2 engages high-openness staff in personality-sensitive innovation workshops to develop novel classification methods. Assessments must combine theoretical knowledge and practical skills, complemented by incentive mechanisms such as “Medical Waste Excellence Banner” awards for departments and “Environmental Guardian” awards for individuals to promote active engagement. This initiative requires evaluation via a 24-month multicenter cluster-randomized trial using digital tracking to monitor behavioral changes and system-level adoption rates.

Per the “weakest link” principle (35), training should prioritize male medical staff demonstrating lower compliance, while introverted staff should be encouraged to participate in group activities to improve knowledge acquisition. Grouping staff with similar personality traits can optimize daily waste classification practices, supplemented by platforms for sharing self-management experiences to strengthen attitudes. Despite established correlations between conscientiousness and improved waste segregation in healthcare settings (36, 37), this study found no significant association—potentially due to social desirability bias or fear of penalties. Nevertheless, hospitals should develop targeted interventions leveraging conscientiousness-related motivations (e.g., emphasizing duty or systematic protocols) and deploy competent staff (with accurate knowledge and positive attitudes) as mentors to enhance overall segregation practices. Administrators should utilize conscientiousness-driven motivations (e.g., emphasizing systematic protocols) for targeted interventions, even amid discrepant findings potentially attributable to penalty fears or normative response biases.

## Data Availability

The raw data supporting the conclusions of this article will be made available by the authors, without undue reservation.
